# The impact of scan time on dynamic $^{68}{\textrm{Ga}}$-FAPI-04 total-body PET parametric imaging generated by deep learning models

**DOI:** 10.1186/s40658-026-00895-z

**Published:** 2026-06-03

**Authors:** Jidong Han, Yu Liu, Meiyong Huang, Lingxin Chen, Yunlong Gao, Xinlan Yang, Jianjun Liu, Dong Liang, Zhanli Hu, Ruohua Chen, Bo Yuan

**Affiliations:** 1 Research Center for Medical AI, Shenzhen Institutes of Advanced Technology, Chinese Academy of Sciences, Shenzhen, 518055 Guangdong China; 2 Key Laboratory of Biomedical Imaging Science and System, Chinese Academy of Sciences, State Key Laboratory of Biomedical Imaging Science and System, China, Shenzhen Institutes of Advanced Technology, Chinese Academy of Sciences, Shenzhen, 518055 Guangdong China; 3 Central Research Institute, United Imaging Healthcare Group, Shanghai, 201800 China; 4 Department of Nuclear Medicine, Ren Ji Hospital, School of Medicine, Shanghai Jiao Tong University, Shanghai, 200127 China; 5 Department of Science and Education, The Fourth People’s Hospital of Shenzhen, Shenzhen, 518118 Guangdong China

**Keywords:** Scan time, Dynamic $^{68}{\textrm{Ga}}$-FAPI-04 total-body PET parametric imaging, Dynamic PET, Static PET, Deep learning models

## Abstract

**Purpose:**

To date, some studies have employed deep learning techniques to directly generate dynamic positron emission tomography (PET) parametric images from static PET. Compared with traditional methods, this approach requires only a single PET/computed tomography (CT) scan. However, current methods tend to employ static PET images captured at fixed scanning times without considering whether static PET images acquired at different scanning times impact the quality of the dynamic PET parametric images generated via deep learning.

**Methods:**

A single-frame image from dynamic $^{68}{\textrm{Ga}}$-FAPI-04 total-body PET can actually be regarded as a static PET image acquired during that specific period of the PET/CT scan. We extracted 5 frames of dynamic $^{68}{\textrm{Ga}}$-FAPI-04 total-body PET at equal intervals, specifically frames 50, 76, 80, 86, and 92. Each frame of the dynamic $^{68}{\textrm{Ga}}$-FAPI-04 total-body PET images was subsequently input into the deep learning model to obtain dynamic $^{68}{\textrm{Ga}}$-FAPI-04 total-body PET parametric images. By comparing and analyzing the quality of dynamic PET parametric images, we attempted to determine the optimal scan time for static PET.

**Results:**

The experimental results revealed that the dynamic $^{68}{\textrm{Ga}}$-FAPI-04 total-body PET parametric image generated from the 58th frame of the dynamic PET image via the deep learning model exhibited the poorest performance. This may be because the diffusion of the radioactive tracer was not stable at the time of PET/CT scanning in frame 58, but it was relatively stable from frames 70–92.

**Conclusion:**

Static PET images acquired at different scanning times do indeed affect the quality of the dynamic $^{68}{\textrm{Ga}}$-FAPI-04 total-body PET parametric images generated via deep learning. Specifically, when the radioactive tracer is unstable during the early stage of scanning, the quality of the dynamic $^{68}{\textrm{Ga}}$-FAPI-04 total-body PET parametric images generated from static PET images via deep learning appears to be inferior.

## Introduction

Positron emission tomography (PET) is an advanced nuclear medicine imaging technology that is widely used in the diagnosis and treatment of tumors [1–3]. On the basis of the axial field-of-view (FOV), PET/CT scanners can be divided into short-axial FOV PET, long-axis FOV PET and total-body PET [4, 5]. Total-body PET enables single-bed-position whole-body imaging, such as 2-m PET/CT from the United Imaging Healthcare Group in Shanghai. Compared with other axial field-of-view PET methods, total-body PET is significantly superior in imaging efficiency, image quality and sensitivity [6, 7].Considering these advantages, this paper is focused on total-body PET images. Note that unless otherwise specified, the PET images mentioned in this paper refer to total-body PET images.

At present, common PET images include static PET and dynamic PET [8–10]. Static PET is obtained after the radioactive tracer has stabilized, whereas dynamic PET involves continuous PET/CT scanning of the patient starting from the injection of the radiopharmaceutical. Static PET has the advantages of fast imaging speed and simple operation, but it also has the disadvantage of providing limited information. For example, it can provide only the distribution of radioactive tracers at a specific time point rather than dynamic information that changes over time. Alternatively, although dynamic PET has the advantages of providing more physiological information and reducing uncertainties, it also has the disadvantages of longer scanning times and higher costs.

In contrast, dynamic PET parametric images are a series of images obtained through mathematical modeling and analysis of dynamic PET data, which provide important information regarding the kinetic behavior of radioactive tracers in the body [11–13]. Compared with static PET, this imaging approach can provide quantitative information such as uptake constants ($K_1$, $K_i$, etc.). Compared with dynamic PET, it reduces the amount of data and improves the diagnostic efficiency of clinical doctors by extracting key quantitative indicators. Furthermore, the dynamic PET parametric images are highly important for understanding the mechanisms of biological processes, assessing disease states, and guiding treatment decisions [14–18]. Francesco et al. applied dynamic parameters to evaluate brain gliomas in children [14]; Thomas et al. used multiparametric dynamic $^{18}{\textrm{F}}$-fluoroethyl-tyrosine PET-MRI for diagnosing glioma recurrence [15]; Huang et al. utilized parameter $K_i$ for diagnosing nasopharyngeal carcinoma [16]; Chen et al. applied parametric imaging for diagnosing pancreatic cancer and gastric cancer [17]; and Valeria et al. used parametric imaging for diagnosing triple-negative breast cancer [18]. However, dynamic PET parametric imaging requires continuous PET/CT scanning of patients for an extended period, and to reduce artifacts, patients are typically required to maintain the same position throughout the scanning process. This significantly increases the imaging time for dynamic PET parametric imaging and has become a major obstacle hindering its development and clinical application [19].

To address the issue of slow dynamic PET parametric imaging, many scholars have conducted exploratory research aimed at reducing scan times [20–23]. Wang et al. employed a dual-time-window (DTW) protocol for whole-body $^{18}{{ \mathrm F}}$-FDG PET dynamic scanning to obtain multiple kinetic parameters [20]. Wang et al. utilized dynamic PET scans at 0*∼ *6 min and 60*∼ *75 min to acquire dynamic PET parametric images [21]. Wu et al. obtained dynamic PET $K_i$ parametric images via two 5-minute PET scans [22]. Moreover, Samimi et al. accurately estimated the metabolic uptake rate constant $K_i$ through a clinically feasible dual time-point (DTP) method [23]. With the rapid development of deep learning, this method has achieved remarkable accomplishments in many fields [24–28]. To realize fast dynamic PET parametric imaging, a few scholars have attempted to address the issue of slow dynamic PET parametric imaging from the perspective of deep learning [29–31]. For instance, Miao et al. utilized U-Nets to generate $K_i$ parametric images from static standardized uptake value (SUV) ratio (SUVR) images [29]. Huang et al. employed a U-Net to generate total-body $K_i$ parametric images from static PET [30]. Wang et al. applied an improved CycleGAN to convert static PET images into dynamic total-body PET parametric images [31].

**Fig. 1:**
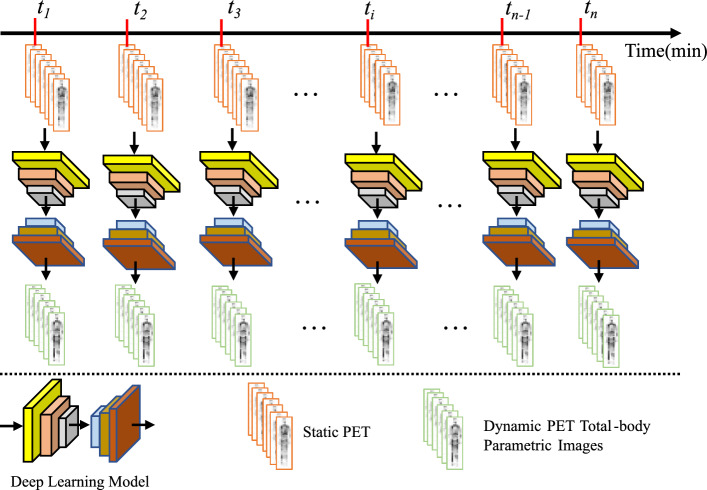
Effect of static PET images obtained at different scanning times on generating dynamic total-body PET parametric images

Directly converting static PET images to dynamic PET parametric images via deep learning methods is equivalent to obtaining dynamic PET parametric images with only a single PET/CT scan. This approach bypasses the need for lengthy continuous dynamic PET/CT scans, effectively compressing the dynamic PET/CT scan time to its absolute minimum. However, these studies were focused only on conventional static PET and did not explore whether static PET images acquired at different PET/CT scan durations affect the quality of dynamic PET parametric images generated by deep learning methods. To solve this problem, we investigated whether static PET images acquired at different PET/CT scan durations affect the quality of generated dynamic $^{68}{\textrm{Ga}}$-FAPI-04 total-body PET parametric images. Figure 1 illustrates the research motivation of this paper. We also explored the influence of different deep learning methods (such as cold diffusion [32], pix2pix [33] or RED-CNN [34]) on generating dynamic $^{68}{\textrm{Ga}}$-FAPI-04 total-body PET parametric images. We attempted to find the optimal deep learning approach and the best static PET/CT scan time for this method. Note that during the experiment, we treated dynamic single-frame PET images with different scanning times as static PET images.

## Materials and methods

### Participants

Our data were provided by Shanghai Renji Hospital. The patients in the dataset were diagnosed with gastric cancer (4 cases), pancreatic cancer (2 cases), or pancreatic lesions (4 cases). All dynamic $^{68}{\textrm{Ga}}$-FAPI-04 total-body PET data were obtained through continuous scanning of patients via the United Imaging Healthcare Group total-body PET/CT uEXPLORER between April and November 2021. To ensure the reliability of the experiment, we employed a five-fold cross-validation method, which involved dividing all patients into five groups for cross-validation of the experimental results, as shown in Table 1.

**Table 1:** Patient information for each group in 5-fold cross-validation (mean±standard deviation)

Group number	1	2	3	4	5
Number of participants	2	2	2	2	2
Gender (male/female)	1/1	0/2	2/0	1/1	0/2
Age* (year)	43.500±7.500	49.500±11.500	47.500±5.500	60.500±2.500	43.000±6.000
Weight* (kg)	47.250±2.750	45.500±4.500	64.500±9.500	56.500±5.500	53.000±1.000
Height* (m)	1.610±0.060	1.635±0.055	1.595±0.015	1.755±0.005	1.685±0.115
Total dose* (MBq)	125.116±29.804	99.382±4.255	127.861±22.952	117.512±6.105	102.102±7.641
Disease type	gastric cancer, pancreatic lesion	gastric cancer	gastric cancer, pancreatic lesion	pancreatic cancer, pancreatic lesion	pancreatic cancer, pancreatic lesion
Scanner	United Imaging Healthcare Group uEXPLORER				

### Dynamic PET and DV parametric image acquisition

**Fig. 2:**
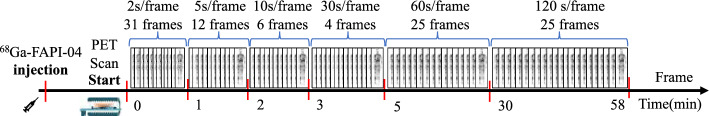
Dynamic $^{68}{\textrm{Ga}}$-FAPI-04 total-body PET scanning protocol

The dynamic $^{68}{\textrm{Ga}}$-FAPI-04 total-body PET scanning protocol is illustrated in Fig. 2. After the injection of $^{68}{\textrm{Ga}}$-FAPI-04, patients underwent dynamic PET continuous scanning for 58 min. The specific scanning protocol was as follows: 0–1 min: 31 frames, 2 s/frame; 1–2 min: 12 frames, 5 s/frame; 2–3 min: 10 s/frame, 6 frames; 3*∼ *5min: 4 frames, 30 s/frame; 5–30 min: 25 frames, 60 s/frame; and 30–58 min: 25 frames, 120 s/frame. Each patient’s dynamic $^{68}{\textrm{Ga}}$-FAPI-04 total-body PET consists of 92 frames, with each frame containing 673 slices, an interslice thickness of 2.886 mm, and a slice image size of 360*360. The distribution volume (DV) parametric images are derived through the application of a 2T4C (two-tissue four-compartment) model to the dynamic $^{68}{\textrm{Ga}}$-FAPI-04 total-body PET data.

### Our method

Compared with static PET and dynamic PET imaging, dynamic PET parametric imaging offers certain advantages. However, the slow imaging of dynamic PET parametric images has become one of the main obstacles hindering the development and clinical application of the approach. The question of how to rapidly acquire dynamic PET parametric images has consistently been a research hotspot in this field. To address this issue, research studies have been conducted on applying deep learning to fast dynamic PET parametric imaging. The main implementation approach is shown in Eq. (1):

1 $$\begin{aligned} X\overset{ \Phi }{\rightarrow }Y \end{aligned}$$

where *X* represents the static PET image, *Y* represents the dynamic PET parameter image, and *Φ * denotes a learnable deep learning model. Although this method can directly generate dynamic PET parametric images, it also has drawbacks of instability and limited generalizability. Moreover, current studies tend to merely utilize static PET images obtained from fixed-time PET/CT scans without considering whether static PET images from PET/CT scans at different time points affect the quality of dynamic PET parametric images generated by deep learning models. To address this issue, we conducted exploratory research.

**Fig. 3:**
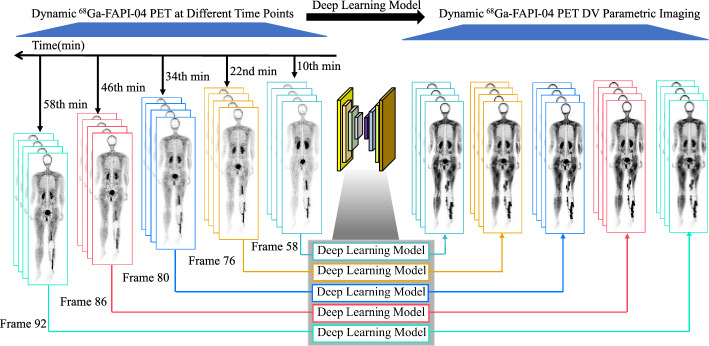
Dynamic $^{68}{\textrm{Ga}}$-FAPI-04 PET total-body DV parametric images generated by dynamic $^{68}{\textrm{Ga}}$-FAPI-04 PET total-body PET at different time points

Our method is illustrated in Fig. 3. A single-frame image of dynamic $^{68}{\textrm{Ga}}$-FAPI-04 total-body PET can actually be regarded as a static PET image obtained from a PET/CT scan performed at that specific time. Considering that the diffusion of radioactive tracers requires time and that the PET/CT scans in the dynamic $^{68}{\textrm{Ga}}$-FAPI-04 total-body PET scanning protocol are unevenly distributed, we did not select images at equal intervals on the basis of frame numbers. Instead, we selected 5 frames from the dynamic $^{68}{\textrm{Ga}}$-FAPI-04 total-body PET at equal time intervals, specifically frames 50, 76, 80, 86, and 92. Then, we input each frame of the dynamic $^{68}{\textrm{Ga}}$-FAPI-04 total-body PET image separately into the deep learning model to obtain the dynamic $^{68}{\textrm{Ga}}$-FAPI-04 total-body PET DV parametric image. By comparing the generated dynamic $^{68}{\textrm{Ga}}$-FAPI-04 total-body PET DV parametric images, we can further analyze and determine the optimal PET/CT scanning time for static PET. Note that the optimal PET/CT scanning time refers to the static PET scan time required for generating dynamic $^{68}{\textrm{Ga}}$-FAPI-04 total-body PET DV parametric images. Additionally, to investigate whether different deep learning models affect the quality of generated dynamic $^{68}{\textrm{Ga}}$-FAPI-04 total-body PET DV parametric images, we designed various deep learning models.

### Deep learning model processing details

To improve the training speed of deep learning models, we resized each slice image to 128*128 pixels by bilinear interpolation during the training process. Considering the specificity of medical images, to prevent the generation of erroneous dynamic $^{68}{\textrm{Ga}}$-FAPI-04 total-body PET DV parametric images, we did not perform image augmentation operations such as rotation on the static PET images. Moreover, to investigate the sensitivity of different deep learning models in generating dynamic $^{68}{\textrm{Ga}}$-FAPI-04 total-body PET parametric images, we employed cold diffusion [32], pix2pix [33], and RED-CNN [34] in our experiments. During the training process of these three models, the Adam optimizer [35] was utilized. Owing to the differences among the models, their learning rates and training epochs were not the same: 2e-5, 1000 epochs (cold diffusion [32]); 2e-4, 2000 epochs (pix2pix [33]); and 1e-5, 800 epochs (RED-CNN [34]). In our experiments, we used an NVIDIA A4000 GPU and 2D axial slices to train all deep learning models. Additionally, both the backbone network structures of cold diffusion [32] and RED-CNN [34] are mentioned in the original paper. For the pix2pix [33] method, we constructed a backbone network consisting of 3 downsampling convolutional blocks (Conv+IN+ReLU), 3 residual blocks (Conv+IN+ReLU+Conv+IN), 2 upsampling convolutional blocks (ConvTranspose+IN+ReLU), and an output layer (Conv+Sigmoid).

To validate the hypothesis presented in this paper, we utilized three deep learning methods: cold diffusion [32], pix2pix [33], RED-CNN [34]. Currently, generative models in deep learning primarily fall into three main categories: diffusion models, generative adversarial networks(GANs), and U-Net. We chose cold diffusion [32] (based on diffusion model), pix2pix [33] (based on GANs), and RED-CNN [34] (based on U-Net) for the following reasons: (1) Comprehensive validation: By choosing one model from each category, we aimed to provide a more comprehensive validation of the three main types of deep learning generative models; (2) Enhanced reliability and persuasiveness: This approach strengthens the reliability and persuasiveness of our experiments by covering a broader spectrum of modeling techniques. Additionally, we chose these models for their specific strengths: (1) Cold diffusion[32] extends beyond the Gaussian noise limitations of traditional diffusion models, offering a more generalized approach; (2) Pix2pix [33] has long been a representative model among GAN-based generative models; (3) RED-CNN [34] is specifically tailored for medical image generation, making it highly relevant to our study.

### Evaluation method

We used the evaluation metrics peak signal-to-noise ratio (PSNR) [36], structural similarity index measure (SSIM) [37], and mean squared error (MSE) to assess the generation of dynamic $^{68}{\textrm{Ga}}$-FAPI-04 total-body PET DV parametric images from dynamic PET images of different frames via deep learning methods. They can be, respectively represented by Eqs. (2–4):

2 $$\begin{aligned} PSNR = 20{\log _{10}\left( \frac{max(x)}{\sqrt{MSE}} \right) } \end{aligned}$$

3 $$\begin{aligned} SSIM = {l\left( {x,y} \right) }^{\alpha } \cdot {c\left( {x,y} \right) }^{\beta } \cdot {s\left( {x,y} \right) }^{\lambda } \end{aligned}$$

4 $$\begin{aligned} MSE = \frac{1}{HW}{\sum \limits _{i = 0}^{H - 1}{\sum \limits _{j = 0}^{W - 1}\left[{x\left( {i,j} \right) - y\left( {i,j} \right) } \right]^{2}}} \end{aligned}$$

where *x* and *y* represent two images; *max*(*x*) denotes the maximum pixel value of *x*; *H* and *W* represent the height and width of image *x* or *y*, respectively; *x*(*i*, *j*) and *y*(*i*, *j*) denote the pixel values of images *x* and *y* at coordinate (*i*, *j*), respectively; *l*(*x*, *y*), *c*(*x*, *y*), and *s*(*x*, *y*) represent the luminance, contrast, and structural comparisons between images *x* and *y*, respectively.

When the quality of the dynamic $^{68}{\textrm{Ga}}$-FAPI-04 total-body PET DV parametric images generated via deep learning is high, the PSNR and SSIM values between the generated images and real images are also greater. This means that the difference between them is smaller; i.e., the MSE value is lower. Conversely, if the quality of the generated images is poor, then the PSNR and SSIM values between the generated images and real images will be lower, and the gap between them will be larger; i.e., the MSE value will be greater. However, this does not necessarily mean that they are consistent. For example, even when the PSNR is high, the SSIM may not be high.

**Fig. 4:**
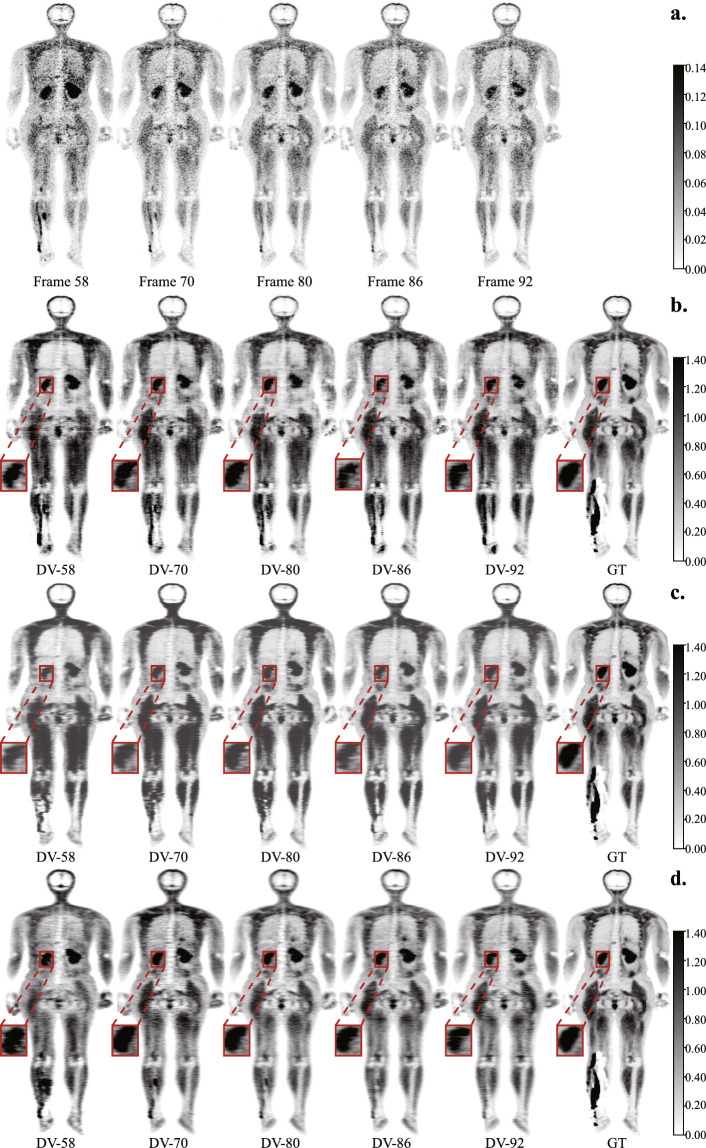
Comparison of the results for the 76th slice in the coronal plane. Tissues or organs in the red box: Right kidney

## Result analysis

### Visualization results analysis

**Fig. 5:**
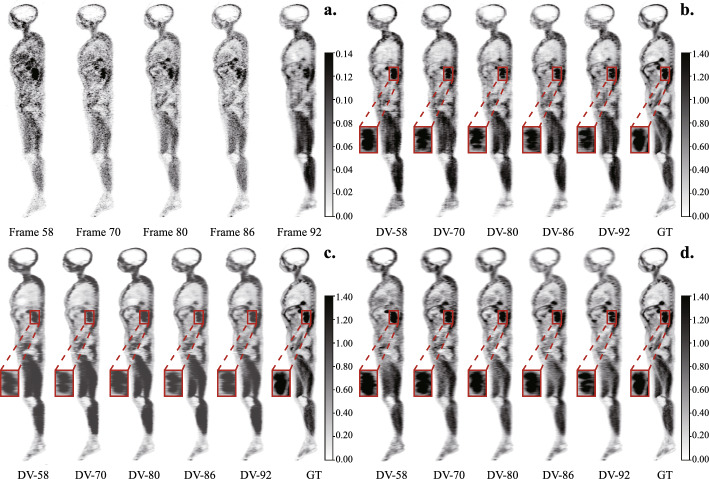
Comparison of the results for the 76th slice in the sagittal plane. Tissues or organs in the red box: Left kidney

**Fig. 6:**
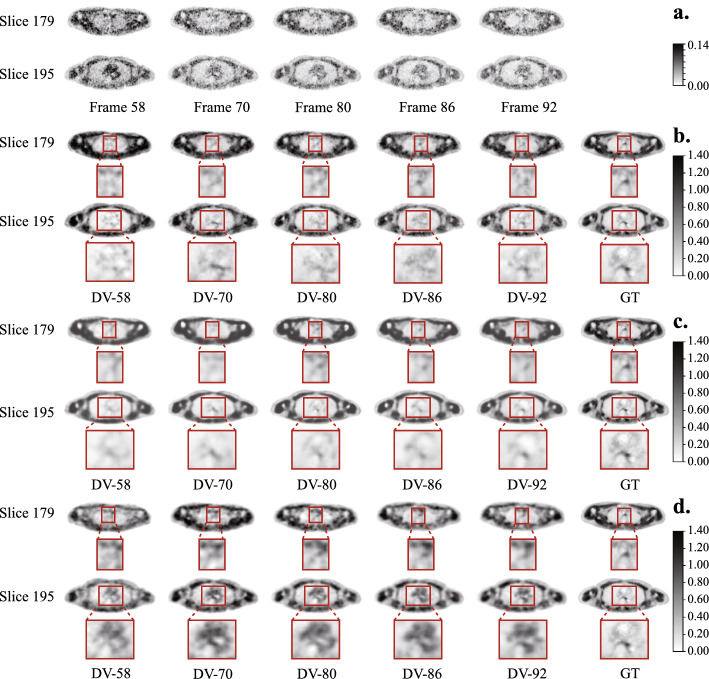
Comparison of the results for the axial planes. Tissues or organs in the red box: Slice 179: Lymph nodes in the 2 L region of the mediastinum; Slice 195: Heart

**Fig. 7:**
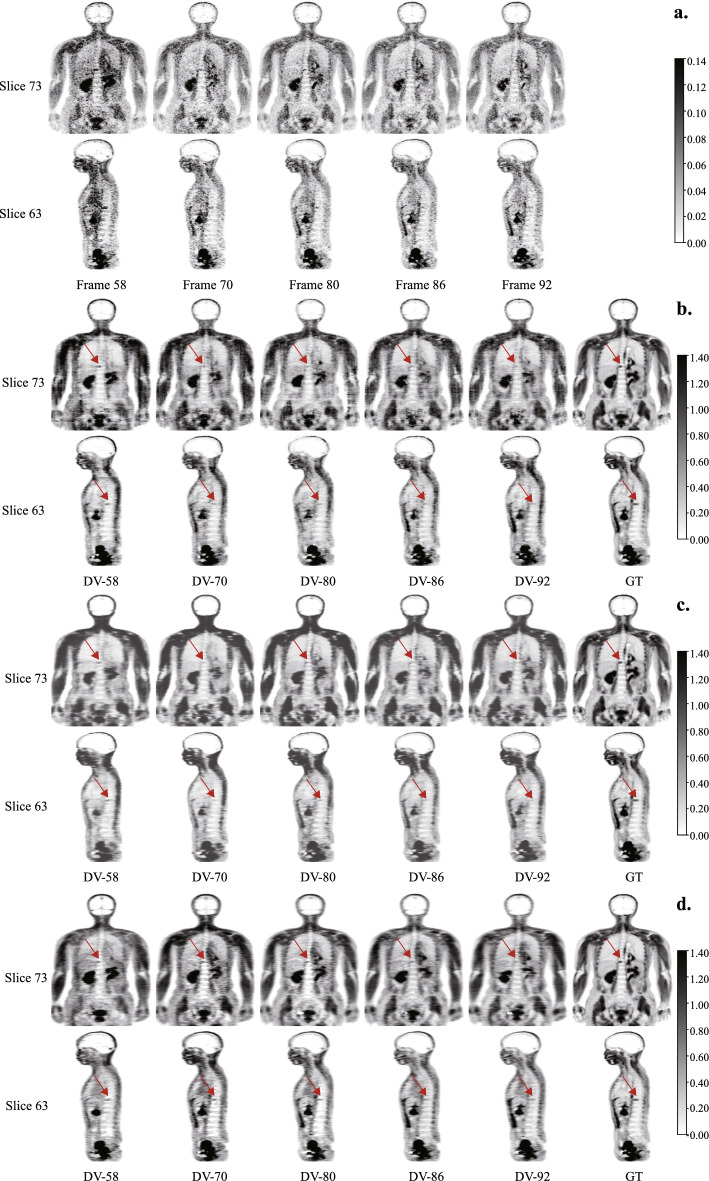
Lesion analysis. Radioactive concentration is visible in the thoracic vertebrae, and clinicians suspect bone metastasis

In this section, we present the coronal, sagittal, and axial results of dynamic $^{68}{\textrm{Ga}}$-FAPI-04 total-body PET DV parametric images generated by different deep learning methods from dynamic PET of different frames, as shown in Figs. 4, 5, 6, and 7. In addition, Figs. 4, 5, 6, and 7 (a) represents the dynamic PET of different frames; (b), (c), and (d) represent the results of dynamic $^{68}{\textrm{Ga}}$-FAPI-04 total-body PET DV parametric images generated by cold diffusion [32], pix2pix [33], and RED-CNN [34], respectively; ’DV-n’ indicates that the image is generated from frame n of dynamic PET through deep learning, such as ’DV-58’, which means that the image is generated from the 58th frame of dynamic PET through deep learning.

From a visual perspective, the image quality of DV-58 is significantly inferior to that of other images in terms of coronal, sagittal, or cross-sectional views. This finding also indicates that the PET/CT scan time indeed affects the quality of the dynamic PET parameters generated via deep learning. From the 58th frame of dynamic PET, we can also observe significant differences compared with other frames in terms of the coronal, sagittal, and axial planes. Conversely, for the 70th, 80th, 86th and 92nd frames of dynamic PET, there are no significant visual differences. Additionally, the quality of the dynamic $^{68}{\textrm{Ga}}$-FAPI-04 total-body PET DV parametric images generated from the 70th, 80th, 86th, 92nd frames of dynamic PET via various deep learning methods is also quite similar.

Contrary to Figs. 4, 5, and 6, Fig. 7 demonstrates the lesion analysis. We can see that the quality of the generated lesion areas is significantly influenced by the deep learning method used. The lesion areas generated by RED-CNN [34] are more distinct, while those generated by cold diffusion [32] and pix2pix [33] methods are more blurred. In the RED-CNN [34] method, the results for DV-70 and DV-80 are better, which is consistent with the previous analysis. Surprisingly, in the cold diffusion [32] and pix2pix [33] methods, the DV-58 result is better than the others. This indicates that scan time also affects the lesion areas in the generated parametric images. As shown in Fig. 7, the tumor lesions appear very clear in the real dynamic $^{68}{\textrm{Ga}}$-FAPI-04 total-body PET DV parametric images, whereas the tumor lesion regions generated by deep learning methods are not fully reproduced and generally exhibit lighter coloration in these areas. This indicates that deep learning methods still have certain limitations in generating tumor lesion regions.

### Overall experimental results analysis

**Fig. 8:**
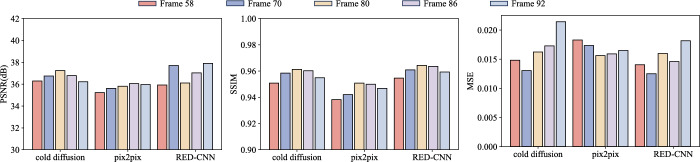
Comparison of overall experimental results

Fig. 8 shows the overall experimental results and corresponds to Tables 2, 3, and 4. Note that bold values in Tables 2-4 denote the optimal values of evaluation metric. From Fig. 8, we can see that the results of cold diffusion [32] and RED-CNN [34] are similar in terms of the SSIM evaluation metric. We believe this is primarily due to the fact that both models are highly suitable for the task of generating dynamic PET parametric images, and secondly, because we applied identical data preprocessing steps for both methods. Pix2pix [33] exhibits poorer performance. However, our focus is not on these aspects. Instead, we are concerned with the following:

- Whether there are differences in the dynamic parameter images generated from different frames of dynamic PET using the same deep learning method;
- Whether different deep learning methods are sensitive to different frames of dynamic PET images.

Based on the results from cold diffusion [32], it can be observed that the results for each frame are not identical in terms of PSNR, SSIM, and MSE metrics, whereas the 80th frame yields the best outcomes. From the pix2pix [33] results, the 58th frame continues to show the worst performance, significantly lower than the other frames, while the results for the 80th and 86th frames are similar, with the 80th frame performing slightly better. It can be seen from the experimental results of RED-CNN [34] that the overall effect of the 58th frame is still the worst, but the performance of other frames is not consistent and scattered in PSNR/SSIM/MSE metrics. This actually indicates that different frames of dynamic PET images have a significant impact on the dynamic $^{68}{\textrm{Ga}}$-FAPI-04 total-body PET DV parameter images generated by deep learning methods. In addition, the variation in the quality of the dynamic PET images obtained by inputting the same frame into different deep learning models is also considerable, suggesting that different deep learning models are also sensitive to different frames of the dynamic PET images.

**Table 2:** Comparison of PSNR/SSIM/MSE for DV parametric images generated from different frames of dynamic PET using the cold diffusion [32]

Evaluation metrics	Frame number	Total	Group 1	Group 2	Group 3	Group 4	Group 5
[2]PSNR(dB)	58	36.293 ±14.530	39.033±15.847	34.290±11.088	38.537±16.315	34.600±10.717	35.006±16.716
	70	36.741±13.031	39.821 ±15.089	$\pmb {35.755\pm 13.181}$	37.600±12.348	$\pmb {35.996\pm 10.291}$	34.534±13.140
	80	$\pmb {37.234\pm 14.300}$	39.812±15.313	34.798±14.641	$\pmb {40.159\pm 17.774}$	35.895±8.240	35.506±12.799
	86	36.786±13.990	40.150±14.984	34.845±14.968	39.105±15.463	34.897±10.547	34.933±12.336
	92	36.218±14.076	$\pmb {40.228\pm 15.721}$	34.295±16.299	36.913±12.754	33.762±10.087	$\pmb {35.891\pm 13.678}$
[2]SSIM($10^{-2}$)	58	95.083±3.806	96.645±2.363	95.455±2.643	96.522±2.679	94.192±3.910	92.600±5.078
	70	95.839±3.404	97.252±1.872	$\pmb {96.290\pm 2.437}$	96.893±2.323	95.775±2.922	92.985±4.807
	80	$\pmb {96.127\pm 3.345}$	97.156±2.096	95.672±3.323	$\pmb {97.267\pm 2.387}$	$\pmb {96.336\pm 2.106}$	$\pmb {94.205\pm 4.905}$
	86	96.027±3.424	$\pmb {97.537\pm 1.615}$	95.682±3.241	97.244±2.161	95.674±2.632	93.997±5.078
	92	95.491±3.791	97.423±1.702	94.965±3.971	96.282±3.150	94.680±3.136	94.106±5.120
[2]MSE($10^{-2}$)	58	1.481±1.509	1.010±0.898	$\pmb {1.817\pm 1.755}$	1.170±1.066	1.359±1.505	2.048±1.822
	70	$\pmb {1.304\pm 1.448}$	0.806±0.750	2.004±2.194	1.039±1.027	0.915±0.792	1.757±1.502
	80	1.623±2.628	0.835±0.785	3.944±4.807	1.031±1.092	$\pmb {0.786\pm 0.712}$	$\pmb {1.519\pm 1.432}$
	86	1.728±2.708	$\pmb {0.779\pm 0.793}$	3.941±4.890	$\pmb {1.026\pm 1.087}$	1.096±0.923	1.796±1.841
	92	2.142±3.878	0.816±0.825	5.524±7.329	1.364±1.443	1.370±1.103	1.638±1.692

“Total” represents the overall result of the five-fold cross-validation, while "Group i" denotes the result for the i-th (i=1, 2, 3, 4, 5) group. (mean±standard deviation)

**Table 3:** Comparison of PSNR/SSIM/MSE for DV parametric images generated from different frames of dynamic PET using the pix2pix [33]

Evaluation metrics	Frame number	Total	Group 1	Group 2	Group 3	Group 4	Group 5
[2]PSNR(dB)	58	35.231±10.371	35.214±9.531	37.082±11.987	35.535±11.241	34.110±8.966	34.211±9.523
	70	35.615±10.271	36.114±10.295	37.285±11.731	35.743±10.915	34.579±7.999	34.355±9.747
	80	35.813±10.325	$\pmb {36.590\pm 9.942}$	35.916±8.740	36.320±11.080	$\pmb {35.008\pm 9.238}$	$\pmb {35.231\pm 12.159}$
	86	$\pmb {36.050\pm 11.183}$	36.185±9.155	$\pmb {38.169\pm 12.994}$	$\pmb {36.912\pm 13.393}$	34.445±8.044	34.540±10.886
	92	35.964±11.127	36.020±9.325	37.873±12.556	36.837±12.929	34.620±8.861	34.469±10.971
[2]SSIM($10^{-2}$)	58	93.821±13.856	94.065±15.209	95.257±12.395	94.151±15.477	93.291±12.515	92.341±13.197
	70	94.207±14.132	94.516±15.686	95.402±12.904	94.512±15.489	94.112±12.675	92.492±13.452
	80	$\pmb {95.081\pm 11.812}$	$\pmb {95.672\pm 12.919}$	95.914±10.577	$\pmb {95.510\pm 13.196}$	$\pmb {94.924\pm 10.428}$	$\pmb {93.385\pm 11.484}$
	86	94.991±12.033	95.437±13.411	$\pmb {96.308\pm 10.296}$	95.280±13.459	94.810±10.683	93.123±11.716
	92	94.681±12.786	95.119±14.130	95.945±11.535	95.200±13.912	94.439±11.285	92.699±12.560
[2]MSE($10^{-2}$)	58	1.829±3.984	1.912±4.195	1.395±3.416	2.009±4.367	1.857±3.959	1.974±3.884
	70	1.736±3.901	1.832±4.263	1.357±3.470	1.873±4.053	1.641±3.662	1.975±3.973
	80	$\pmb {1.561\pm 3.402}$	$\pmb {1.549\pm 3.575}$	1.255±3.086	$\pmb {1.686\pm 3.858}$	1.569±3.289	$\pmb {1.747\pm 3.120}$
	86	1.590±3.436	1.588±3.680	$\pmb {1.176\pm 2.978}$	1.797±3.828	$\pmb {1.520\pm 3.318}$	1.872±3.264
	92	1.649±3.571	1.648±3.776	1.264±3.312	1.725±3.737	1.640±3.596	1.967±3.377

"Total" represents the overall result of the five-fold cross-validation, while "Group i" denotes the result for the i-th (i=1, 2, 3, 4, 5) group. (mean±standard deviation)

**Table 4:** Comparison of PSNR/SSIM/MSE for DV parametric images generated from different frames of dynamic PET using the RED-CNN [34]

Evaluation metrics	Frame number	Total	Group 1	Group 2	Group 3	Group 4	Group 5
[2]PSNR(dB)	58	35.928±15.924	39.185±16.882	30.750±14.498	38.509±16.003	$\pmb {35.740\pm 13.069}$	35.454±17.365
	70	37.708±15.719	$\pmb {40.194\pm 16.217}$	37.589±17.904	41.968±21.597	35.389±7.609	33.398±8.898
	80	36.107±11.823	38.302±10.092	34.917±14.466	38.420±11.855	34.358±11.459	34.537±9.941
	86	37.034±13.915	38.635±11.694	$\pmb {37.674\pm 18.331}$	37.604±12.068	35.310±8.501	$\pmb {35.945\pm 16.434}$
	92	$\pmb {37.906\pm 19.529}$	39.122±13.419	35.063±14.862	$\pmb {44.902\pm 31.861}$	35.298±16.923	35.147±11.472
[2]SSIM($10^{-2}$)	58	95.461±3.719	96.476±2.555	96.211±2.589	96.531±2.731	94.899±3.588	93.187±5.232
	70	96.091±3.312	97.290±1.843	96.367±2.613	97.497±1.745	96.063±2.638	93.242±4.797
	80	$\pmb {96.429\pm 3.186}$	$\pmb {97.675\pm 1.603}$	96.061±3.133	$\pmb {97.800\pm 1.432}$	$\pmb {96.512\pm 2.337}$	$\pmb {94.095\pm 4.674}$
	86	96.360±3.232	97.539±1.612	$\pmb {96.752\pm 2.387}$	97.369±1.772	96.212±2.533	93.930±5.086
	92	95.925±3.517	97.404±1.719	95.923±3.130	97.246±1.903	95.000±3.184	94.053±5.200
[2]MSE($10^{-2}$)	58	1.405±1.699	1.054±1.023	$\pmb {1.408\pm 1.505}$	1.192±1.160	1.325±2.327	2.046±1.943
	70	$\pmb {1.251\pm 1.609}$	0.776±0.721	2.114±2.684	0.811±0.806	$\pmb {0.895\pm 1.037}$	1.658±1.426
	80	1.599±3.034	$\pmb {0.754\pm 0.788}$	3.996±5.714	$\pmb {0.787\pm 0.804}$	1.010±1.705	$\pmb {1.446\pm 1.309}$
	86	1.461±2.161	0.764±0.742	2.696±3.752	0.971±0.932	1.025±1.398	1.846±1.817
	92	1.817±3.122	0.810±0.785	4.015±5.820	1.032±1.038	1.564±1.931	1.663±1.703

"Total" represents the overall result of the five-fold cross-validation, while "Group i" denotes the result for the i-th (i=1, 2, 3, 4, 5) group. (mean±standard deviation)

### Single group experiment result analysis

**Fig. 9:**
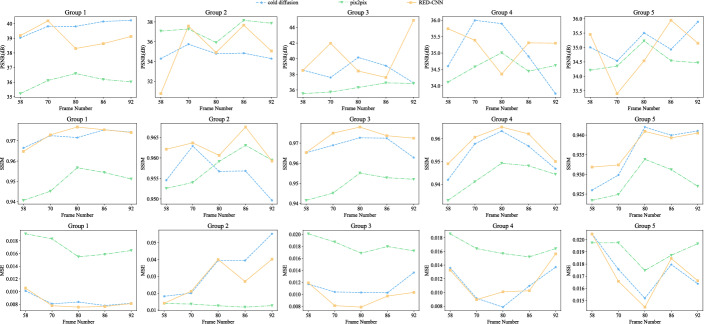
Comparison of experimental results for a single group

Fig. 9 shows the experimental results for a single group. The horizontal axis represents the frame number of the dynamic PET image, and the vertical axis represents the evaluation metrics of the PSNR, SSIM, or MSE. As shown in Fig. 9, the results are almost entirely consistent with the overall results. The specific results in Tables 2, 3, and 4 also indicate that the results for a single group are basically consistent with the optimal values of the overall results. Therefore, the experimental results from a single group also show that there are certain differences in the dynamic PET parametric images obtained by inputting dynamic PET images of different frames into deep learning models, and these differences can be influenced by the PET/CT scan time. Furthermore, from Fig. 9, it can be seen that deep learning models are still relatively sensitive to different frames of dynamic PET images, meaning that the differences in dynamic $^{68}{\textrm{Ga}}$-FAPI-04 total-body PET DV parametric images generated by different deep learning models are still quite significant.

### ROI experimental result analysis

**Fig. 10:**
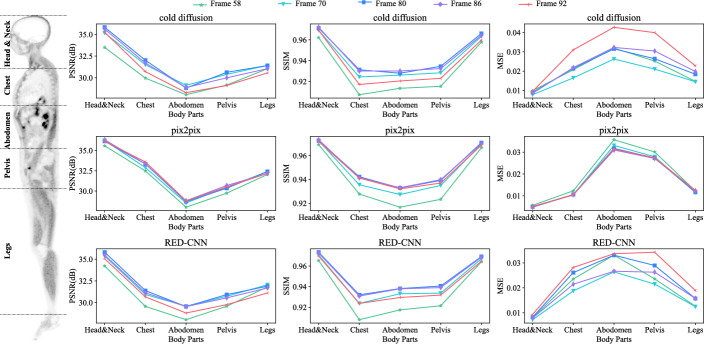
Comparison of PSNR/SSIM/MSE for patient ROI analysis

Fig. 10 presents the experimental results for patient ROIs, where the horizontal axis represents different body parts, such as the head and neck, chest, abdomen, pelvis, and legs, and the vertical axis corresponds to quantitative evaluation metrics. Since nuclear medicine imaging studies of the feet are relatively rare, we did not include quantitative metrics for the feet in our assessment. As shown in Fig. 10, the overall results are basically consistent with those of the previous analysis, indicating that the dynamic $^{68}{\textrm{Ga}}$-FAPI-04 total-body PET DV parametric images obtained from the 58th frame still perform poorly across all the ROIs. Surprisingly, the quality of the dynamic $^{68}{\textrm{Ga}}$-FAPI-04 total-body PET DV parametric images generated by various deep learning methods generally shows a trend of first decreasing and then increasing along the from head to legs in the human body.

**Fig. 11:**
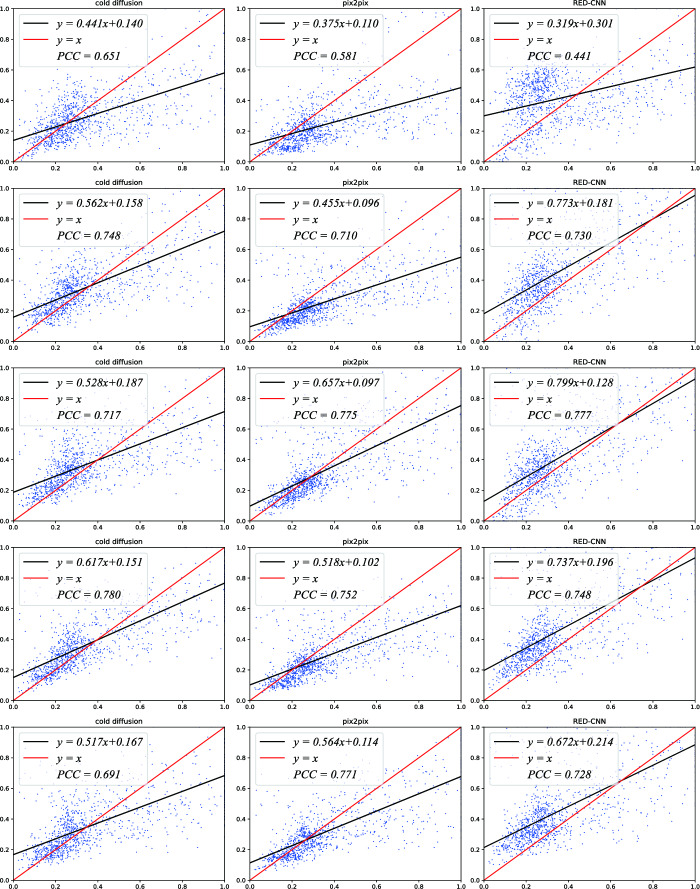
Correlation analysis for tumor ROI

Fig. 11 shows the results of the correlation analysis for tumor ROIs, where rows 1*∼ *5 correspond to the fitting results of tumor ROIs for DV-58, DV-70, DV-80, DV-86, and DV-92, respectively. We first manually annotated the tumor ROIs in the generated dynamic $^{68}{\textrm{Ga}}$-FAPI-04 total-body PET DV parametric images and the ground true images. Then, we used a linear function *y=kx+b* for fitting, with the results shown in Fig. 11. In the figure, the red line *y=x* represents the ideal outcome, indicating that the tumor ROIs in the generated dynamic $^{68}{\textrm{Ga}}$-FAPI-04 total-body PET DV parametric images are exactly the same as those in the ground true images. The black fitting line *y=kx+b* indicates the actual fitting performance (closer proximity to the red line suggests better agreement). The blue points represent the scatter plot of the generated tumor ROIs. The Pearson Correlation Coefficient (PCC) value in the figure indicates the correlation between the tumor ROIs in the generated dynamic $^{68}{\textrm{Ga}}$-FAPI-04 total-body PET DV parametric images and those in the ground true images. A value greater than 0 indicates a positive correlation, and the closer it is to 1, the stronger the positive correlation. As can be seen from Fig. 11, the results do not align very closely with the visual results in Fig. 7. This is because the fitting outcome is determined by the pixel values of the overall ROI in the selected region. From Fig. 11, it can be observed that the cold diffusion method [32] performs better at frame 86, while the pix2pix [33] and RED-CNN [34] methods perform better at frame 80. This indicates that for tumor ROIs in dynamic $^{68}{\textrm{Ga}}$-FAPI-04 total-body PET DV parametric images, the impact of different scanning times on deep learning methods varies. From the results of the correlation analysis, there is still a gap between the tumor ROIs of the generated dynamic $^{68}{\textrm{Ga}}$-FAPI-04 total-body PET DV parametric images and those of the real images. Specifically, the best-fit result *y=0.799x+0.128* shows a certain deviation from the ideal fit line *y=x*, and the best PCC value of 0.780 also differs significantly from the ideal value of 1. In future research, we will focus on improving the performance of deep learning methods in generating ROIs such as tumors and other lesions.

The previous analysis revealed that the scan time of dynamic PET affects the parametric images generated by deep learning models and that different deep learning models have varying sensitivities to the scan time of dynamic PET. In both the qualitative and quantitative analyses, the dynamic PET parametric images generated from the 58th frame through the deep learning model showed the latest effects, which may be attributed to the unstable diffusion of radioactive tracers during the 58th frame PET/CT scan. In contrast, during the PET/CT scans from the 70th to 92nd frames, the diffusion of radioactive tracers had already stabilized relatively.

## Discussion

Some studies have demonstrated that dynamic PET parametric images hold significant value in tumor diagnosis. It exhibits higher contrast and specificity, enabling clearer visualization of small lymph node metastases or distant metastatic lesions. This facilitates more accurate tumor staging, directly influencing the selection of treatment strategies. Furthermore, evaluating specific biological characteristics of tumors through dynamic PET parametric images assists in selecting the most effective therapeutic drugs. However, how to quickly and conveniently obtain dynamic PET parametric images remains a challenge. Currently, many researchers are attempting to use dual-time-point PET/CT scans to acquire dynamic PET parametric images, but this method still requires patients to maintain the same posture for a prolonged period during scanning. Therefore, directly generating dynamic PET parametric images from static PET through deep learning methods becomes a preferable choice. We believe it this approach is feasible primarily because of the following points:

- Both static PET and dynamic PET parametric images are of the same patient, meaning that they share the same anatomical basis.
- Both static PET and dynamic PET parametric images are types of PET images, so they are inherently similar.
- Static PET and dynamic PET parametric images can be considered two relatively similar image domains, and a mapping relationship may exist between them. Deep learning is currently the most suitable technology for automatically discovering such mapping relationships.

In previous studies, dynamic PET parametric images were generated via deep learning techniques from static PET. However, these studies often relied on static PET scans acquired at fixed time points. This prompts the following question: does the input of static PET acquired at different scan times into the deep learning model affect the quality of the generated parametric images? This study revealed that static PET images acquired at different scan times do indeed affect the quality of the generated dynamic parametric images.

In the experimental process, we employed 2D deep learning models instead of 3D models, primarily due to two advantages of the 2D models. First, in terms of computational efficiency and feasibility, dynamic whole-body PET data is extremely voluminous. Training 3D models demands exceptionally high GPU memory and is very time-consuming. In contrast, 2D models utilize limited computational resources much more efficiently, enabling us to complete extensive comparative and cross-validation experiments within a reasonable timeframe. This was crucial for the core objective of this study: investigating the impact of static PET images from different time points on the generation of dynamic PET parametric images by various deep learning methods. Second, 2D models have fewer parameters, which helps reduce the risk of deep learning overfitting−a significant consideration given the limited experimental dataset−and contributes to more stable training outcomes. Therefore, this choice represents a pragmatic consideration aimed at balancing computational cost, experimental iteration speed, and model stability during this exploratory research phase. Compared to 3D models, which can capture three-dimensional neighborhood information, the primary limitation of 2D models lies in the loss of spatial context perpendicular to the slice plane. This may result in insufficient capability for extracting features that depend on the continuity of three-dimensional anatomical structures, potentially affecting the local accuracy of parametric images in the coronal and sagittal planes. However, since the quantitative metrics in our experiments focus on the axial plane, the local impact on the coronal and sagittal planes does not affect our quantitative analysis.

However, our research also has certain limitations. While we have verified that static PET scans acquired at different time points do indeed impact the quality of the parametric images generated by deep learning, we have not yet determined the optimal scan time for acquiring dynamic PET parametric images. Additionally, owing to the difficulty in acquiring PET images, we utilized only dynamic $^{68}{\textrm{Ga}}$-FAPI-04 total-body PET DV parametric images in our study and did not validate our conclusions via other parametric images. Furthermore, since $^{68}{\textrm{Ga}}$-FAPI-04 is a new drug, the number of patients we have collected is relatively small. Next, we will attempt to construct a new method to determine the best scanning time of static PET for generating dynamic PET parameter images through deep learning technology. We will collect as many dynamic PET images and different types of dynamic PET parametric images as possible to further verify our hypothesis. In addition, the limitations of the 2D model imply that the dynamic $^{68}{\textrm{Ga}}$-FAPI-04 total-body PET DV parametric images generated in this study may not yet meet the standards for direct use in refined clinical decision-making−such as the absolute quantitative assessment of small lesions−in terms of absolute quantitative accuracy and spatial consistency. Nonetheless, this does not diminish the valuable insight this study provides for determining the "optimal acquisition time window." To translate this finding into a clinical tool, future work must further improve the generation quality of deep learning models to ensure that the produced parametric images possess sufficient anatomical coherence and quantitative reliability.

## Conclusion

To address the issue of slow imaging in dynamic PET parametric images, one approach is to directly generate dynamic PET parametric images from static PET scans via deep learning techniques. Compared with dual-time-point PET/CT scans, this method significantly reduces the scan time, essentially compressing the scan time to its maximum efficiency. However, current methods often rely on static PET images acquired at fixed time points without considering whether static PET scans at different time points impact the quality of the parametric images generated via deep learning. To address this question, we designed an experimental protocol to test our hypothesis. That is, do static PET images acquired at different scan times affect the quality of the parametric images generated via deep learning? We considered different frames of dynamic PET images as static PET images obtained at various scan times, fed them separately into the deep learning model, and finally evaluated the quality of the resulting dynamic $^{68}{\textrm{Ga}}$-FAPI-04 total-body PET DV parametric images. The experimental results indicate that static PET images acquired at different scan times do indeed influence the quality of dynamic parametric images generated via deep learning. Additionally, the quality of dynamic PET parametric images generated by deep learning from static PET images captured during the early, unstable phase of the radioactive tracer appears to be inferior.

## Data Availability

The datasets generated during and/or analyzed during the current study are available from the corresponding author upon reasonable request.
